# From spirometry to spatial omics in pursuit of asthma endotypes

**DOI:** 10.1002/ctm2.878

**Published:** 2022-09-23

**Authors:** Timothy S. C. Hinks

**Affiliations:** ^1^ Respiratory Medicine Unit and National Institute for Health Research (NIHR) Oxford Biomedical Research Centre (BRC) Nuffield Department of Medicine Experimental Medicine University of Oxford Oxford UK

1

Asthma is heterogeneous, both clinically and pathologically. The importance of this gained traction with the notion of endotypes: subtypes of disease defined functionally and pathologically by a molecular mechanism or by treatment response.^1^ The simplistic view of asthma as a single disease – albeit with a spectrum of severity – led to unified, stepwise treatment algorithms which successfully promoted widespread use of inhaled corticosteroids, and reduced morbidity and mortality, but at the cost of stagnation in research, delaying adoption of personalized medicine,^2^ and nearly causing anti‐interleukin(IL)‐5 biologics to be overlooked. By contrast, cluster analyses of large, carefully phenotyped cohorts improved definitions of clinical phenotypes.^1^, ^3^, ^4^ The last decade has witnessed the clinical efficacy of a treatment paradigm based on identifying and targeting specific phenotypes or ‘treatable traits’.^2^

Nonetheless the basic mechanisms underlying many of these phenotypes remain poorly understood. For instance, severe eosinophilic asthma is driven by IL‐5, yet its cellular source and cause of its persistent overproduction remain unknown. We need to better describe these mechanisms and endotypes, to enable precision medicine and the development of novel therapeutics.

The Severe Asthma Research Program used spirometry and age of onset to define five clinical clusters, which have been reproduced by others, but lacked mechanistic or therapeutic insight (Figure 1).^3^ Haldar's classic cluster analysis described two phenotypes of severe refractory asthma both characterised by discordance between symptoms and eosinophilic airway inflammation and highlighted a poor correlation between the severity of symptoms and the underlying biology.^4^ In pursuit of underlying endotypes, more recent approaches used multimodal immunological assays and topological data analysis to analyse bronchial tissues.^5^ To achieve the power to discover novel phenotypes the U‐BIOPRED initiative analysed over 1000 participants using transcriptomic, proteomic, lipidomic, metabolomic and metagenomic techniques.^6^ One phenotype identified for analysis, and familiar from the clinic, was people with frequent exacerbations.

**FIGURE 1:**
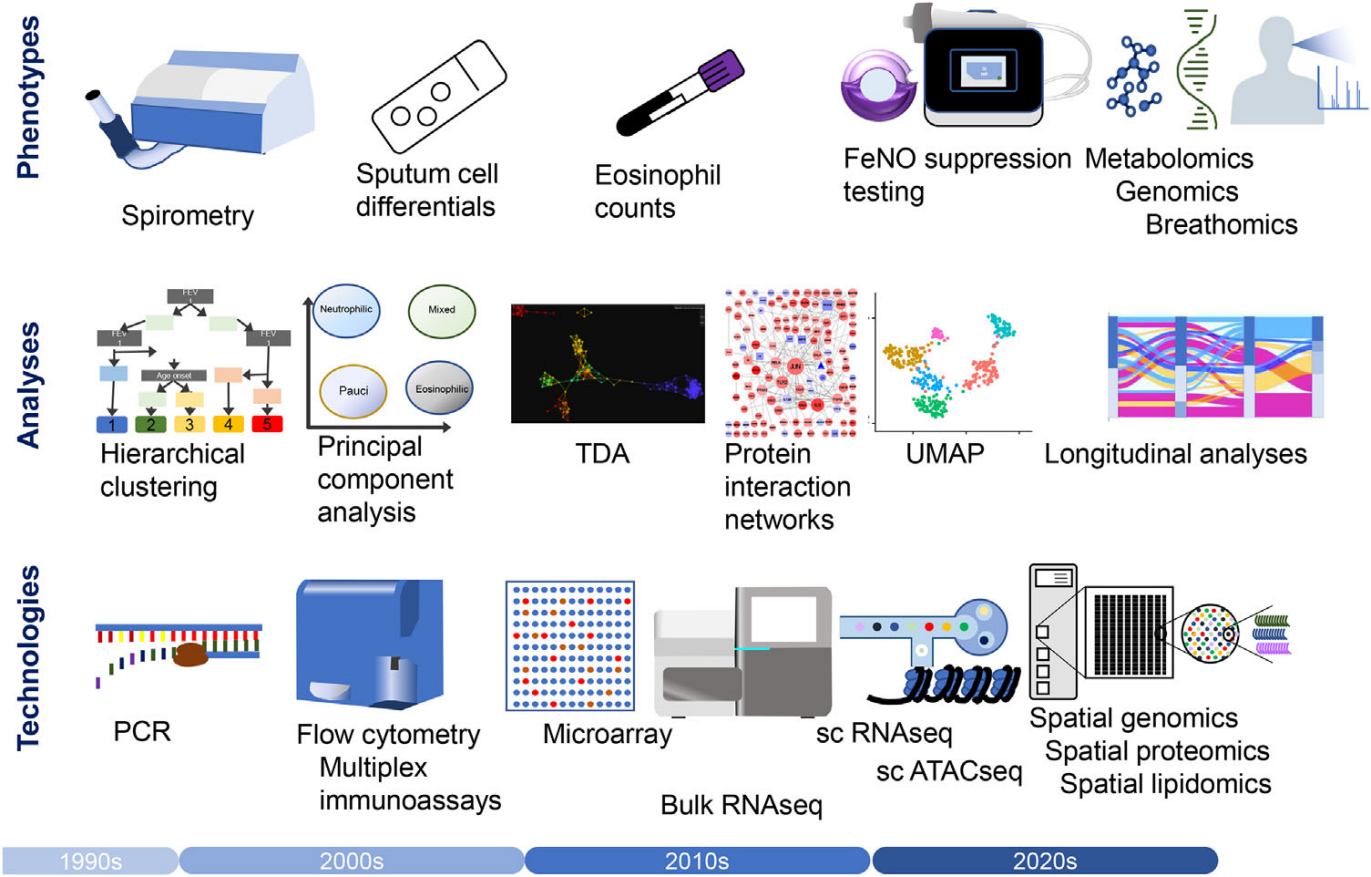
Recent developments in clinical phenotyping, emerging ‘massively‐parallel’ technologies and bioinformatic analyses are accelerating the hunt for endotype‐specific mechanisms in severe asthma. ATAC, assay for transposase‐accessible chromatin; ELISA, enzyme‐linked immunosorbent assay; FeNO, fractional exhaled nitric oxide; PCA, principal component analysis; PCR, polymerase chain reaction; RNA, ribonucleic acid; sc, single cell; seq, sequencing; TDA, topological data analysis; UMAP, uniform manifold approximation and projection

In this issue of *Clin Trans Med* Hoda et al. analysed clinical and transcriptomic features from blood, bronchial and nasal samples from frequent exacerbators (defined as ≥2 exacerbations/ year), who comprised 62% of 317 U‐BIOPRED participants with severe asthma.^7^ This phenotype persisted after a year in 61% of individuals, and was associated with female sex, younger onset, higher symptoms, more SABA use, lower IgE and the presence of sinusitis and eczema. Two important strengths of this analysis were the large sample size of the cohorts and the use of unbiased transcriptomic and proteomic approaches. The bronchial biopsy transcriptomics showed upregulation of CEACAM5 expression in frequent exacerbators, confirming previous findings of its upregulation in asthma.^8^, ^9^ Carcinoembryonic antigen‐related cell adhesion molecule 5 is a cell‐surface protein expressed on airway mucous ciliated airway cells, a cell type more abundant in asthma.^9^ Expression is induced in vitro by IL‐13.^10^ Though a causative role in frequent exacerbations cannot be inferred from correlative studies, it is certainly plausible as CEACAM5 acts an adhesion molecule for bacteria. As with other cell surface molecules, such as ICAM‐1, which can be upregulated by both respiratory bacteria and respiratory viruses, CEACAM5 can also facilitate entry to epithelial cells of viruses including coronaviruses. Thus, upregulated CEACAM5 may increase susceptibility to exacerbations, and this upregulation could be induced either by type II inflammation, or potentially by persistent bacterial infection.

Gene set variation analysis to look at groups of genes, confirmed a previously described increased IL‐13‐induced epithelial signature.^8^, ^11^ This signature is steroid sensitive,^8^ suggesting poor treatment adherence might underlie some frequent exacerbations, although overall this group did not have elevated fractional exhaled nitric oxide (FeNO). Unfortunately, data on corticosteroid adherence were lacking. A T helper‐17 response was postulated as an important mechanism in severe asthma, but has not been borne out in mechanistic studies,^5^ nor by two trials of IL‐17 pathway biologics. Indeed the authors here found suppression of a Th17 signature in frequent exacerbators. Conversely, frequent exacerbations were associated with an eosinophil signature in sputum, but not in tissue.^7^ Given the clear efficacy of anti‐IL‐5 biologics absence of tissue eosinophilia might seem surprising, but is consistent also with immunopathological findings from U‐BIOPRED,^6^ and points to a compartmentalisation of eosinophil dysfunction in asthma.

This analysis is limited by considering frequent exacerbators as a single phenotype. Given the known heterogeneity of severe asthma, this is unlikely and we should expect a range of different mechanisms for frequent exacerbations. This could explain the instability of ‘persistent’ frequent or infrequent exacerbator status in 40% of individuals. In different individuals, frequent exacerbations could be attributable variously to steroid‐resistant type‐2 inflammation, to poor adherence, to eosinophilic inflammation, to deficient type I/III interferon responses, to persistent bacterial infection, and to discordance between symptoms and inflammation.

Future studies will need to tease apart this heterogeneity. Already the MEX study, of exacerbations occurring in mepolizumab recipients, identified two exacerbation phenotypes.^12^ Half displayed high sputum and blood eosinophilia, elevated FeNO and low CRP; the other half sputum neutrophilia, higher CRP and lower FeNO. Steroid resistance and poor adherence are now differentiated objectively using FeNO suppression testing.^13^ This will sharpen clinical phenotyping and has suggested a two‐compartment model where FeNO and blood eosinophils relate to different components and compartments of type‐2 inflammation. FeNO reflects airway type 2 activity and the chemotactic pull to the airways, whereas blood eosinophils reflect the systemic pool of available effector cells and circulating IL‐5.^13^

Given this compartmentalisation, it is clear that immunological spatial location matters. Already the hunt for endotypes has progressed from spirometry and sputum eosinophils to large‐scale analysis of microarray data. Now single‐cell RNA sequencing provides unparalleled information on the behaviour of individual cells. Our ongoing studies are analysing asthma epigenetics at single cell level, with the proteome analysed to a depth of over 2500 different proteins. Emerging technology now enables us to analyse the transcriptome, the proteome and even the lipidome with high spatial resolution to map immune dysfunction within each individual biopsy. This will undoubtedly turbo‐charge the pace of mechanistic research in asthma, but face us with the immense bioinformatic challenge of interpreting and integrating the wealth of data we produce in the quest to understand endotypes.

New technologies are accelerating the hunt for endotype‐specific mechanisms in severe asthma.

## FUNDING INFORMATION

TSCH has received grant support from the Wellcome Trust (211050/Z/18/z, 211050/Z/18/A), from Pfizer and from Sensyne Health.
